# Supplementary material for: Molecular mechanism of lytic polysaccharide monooxygenases
Source: Chem Sci. 2018 Mar 26;9(15):3866–80. doi: 10.1039/c8sc00426a (PMC5935029; doi:10.1039/c8sc00426a)
Supplement: Supplementary file 1 [file SC-009-C8SC00426A-s001.pdf]

Electronic Supplementary Material (ESI) for Chemical Science.
This journal is © The Royal Society of Chemistry 2018

Supporting information for: Molecular mechanism of lytic polysaccharide monooxygenases

Erik D. Hedegård* and Ulf Ryde*

Department of Theoretical Chemistry, Lund University, P. O. Box 124, SE-221 00 Lund, Sweden

E-mail: erik.hedegard@teokem.lu.se; ulf.ryde@teokem.lu.se

Computational procedures and setup

Protein setup

The starting coordinates where taken from the 1.8 Å resolution crystal structure of the LPMO–substrate complex from *Lentinus similis*[S1] (PDB ID 5ACF). The structure is a monomer that contains 250 amino acids and 358 crystal water molecules, amounting to 2216 atoms in total. The structure was collected with a low radiation dose to minimise photoreduction during data collection, which is a well-known problem for metalloproteins[S2] like LPMOs.[S3,S4]

The crystal structure contained one amino-acid residue (Glu235) and ten water molecules with alternative conformations We included in the calculations only the conformation with highest occupation or the first conformation if the occupation numbers were equal. It also contained ten Cl^- ions (one coordinated to Cu) and an N-acetylglucosamin (NAG) group bound to Asn138, which were deleted. Hydrogen atoms were added using the Maestro protein

preparation tools.[S5] For titratable residues (7 arginine, 3 lysine, 7 histidine, 16 aspartate and 5 glutamate residues), Maestro employs the PROPKA program[S6] to estimate pK_a values. The individual residues were visually inspected and their solvent exposure and hydrogen-bond network were assessed. Based on this, we concluded that all arginines and lysines are protonated (+1) and the aspartic and glutamine acids are in their carboxylate form (−1). Three of these charged residues are buried inside the protein, Glu103, Arg140 and Glu142. Glu103 forms an ionic pair with the ammonium group of Lys100, whereas Arg140 and Glu140 from another ionic pair. Therefore, they were considered in the charged forms. The protein contains two cysteine residues (Cys41 and Cys167) that are cross-linked by a disulfide bridge.

Histidine residues have two possible protonation sites and in the following, we denote histidines as HIE ($N^{\epsilon 2}$ protonated), HID ($N^{\delta 1}$ protonated) or HIP (both nitrogens protonated). The N-terminal histidine is a special case, because the imidazole is methylated on the $N^{\epsilon 2}$ atom, whereas $N^{\delta 1}$ coordinates to the Cu ion. For the remaining histidine residues, we employed the protonation states HIP66, HID78, HIP79, HIP122, HID125, HIP131 and HIE147. HIP66 forms hydrogen bonds from $N^{\delta 1}$ to the $O^{\delta 1}$ carboxyl group of Asp72 and from $N^{\epsilon 2}$ to a hydroxy group of the substrate. HID78 coordinates to Cu through $N^{\epsilon 2}$, whereas HIP79 forms a salt-bridge from $H^{\delta 1}$ to the carboxylate group of Asp116 and a hydrogen bond to a crystal water molecule through $H^{\epsilon 2}$. HIP122 forms a hydrogen bond to the carboxyl group of Asp70 through $H^{\delta 1}$ and is exposed on the surface of the protein and was therefore chosen to be doubly protonated. HID125 forms a hydrogen bond to a crystal water molecule from $H^{\delta 1}$ and $N^{\epsilon 2}$ accepts a hydrogen bond from the NH group of the Trp64 side chain. HIP131 is solvent exposed on the surface and was chosen to be doubly protonated (in the crystal structure one of the hydrogen interacts with a Cl^- ion). HIE147 is close to the active site and may participate in the reaction mechanism as the proton donor. The preperation tool in Maestro originally flipped this residue, but we decided instead to employ the HIE form in the conformation obtained in the crystal structure. This form is in agreement with a recent

neutron diffraction study[S7] on a substrate-free structure, which allows $H^{\epsilon 2}$ to interact with the O_2^- ligand in $[CuO_2]^+$. We used this state during setup and equilibration, whereas in the QM/MM calculations we employed sometimes instead the other two states to model various proton-transfer events. With His147 in the HIE state, the total charge of the simulated system in the $[Cu(H_2O)]^{2+}$ (**1**) resting state was -5.

Equilibration and QM/MM setup

The system described above was equilibrated by simulated annealing. Both the equilibration and the QM/MM calculations followed closely our previous investigations[S8] and here we only highlight differences. The previous calculations were carried out on a system without substrate, but in this study, we included a trisaccharide substrate (cf. Figure 1 in the paper). The substrate was described by the glycam.v06 force field,[S9] which is tailored for oligosaccharides. The protein was described with the Amber FF14SB force field[S10] and water molecules with the TIP3P model.[S11] The equilibration was performed on state **1**, which was obtained by replacing the coordinating Cl^- ion in the crystal structure with H_2O.

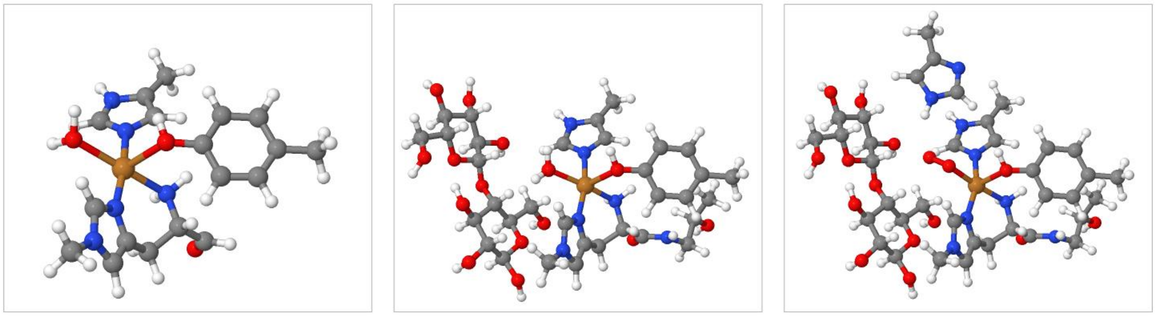

Figure S1: The systems employed for RESP charges (*left*) and two examples of the employed QM systems (*middle* with and *right* without His147; intermediates **1** and **3** are used as examples).

For the equilibration, restrained electrostatic potential (RESP) charges were employed for the metal center and its first coordination sphere (cf. Figure S1) The employed structure was taken from the crystal structure and only the hydrogen atoms were optimized, employing the

TPSS functional[S12] together with the def2-SV(P) basis set.[S13,S14] The electrostatic potential was calculated in points that were sampled with the Merz–Kollman scheme[S15,S16] using default radii for the light atoms and 2 Å for Cu.[S17] They were employed by the RESP program (a part of the AMBER software package) to calculate the charges.

The QM system (system 1) consisted of the copper ion and its first coordination sphere i.e. the imidazole ring of His78 and the phenol ring of Tyr164, both capped with a hydrogen atom replacing C^{α}. The entire His1 residue, which coordinates to Cu through the terminal amino group as well as the imidazole side chain, was included. The neighboring Thr2 residue was included up to the C^{α} atom, which was replaced by a hydrogen atom. In addition, the two first glucose rings of the substrate were also included in system 1, whereas the third glucose unit was described by MM. Thus, for the **1** state, the QM region comprised 111 atoms (see Figure S1, *middle*).

Since the reduction of **1** without substrate has been discussed in several previous computational QM-cluster[S4,S18,S19] and QM/MM studies,[S8] we have also considered the reduced $[Cu(H_2O)]^+$ state (**2**). From the optimised structure of **1** we constructed the $[CuO_2]^+$ (**3**) state by replacing the equatorial H_2O with $O_2^{\bullet-}$. Starting from **3**, we also included His147 within the QM region (Figure S1, *right*) in various states of protonation (HID, HIE or HIP), depending on the intermediate. For reactions where His147 acts as proton donor, we employed the HIP state. For the active intermediates in C−H activation, we studied both HIE and HID forms.

The QM/MM structure optimizations employed the dispersion-corrected TPSS-D3 functional[S12,S20] with Becke–Johnson damping[S21] and the def2-SV(P) basis set.[S13,S14] The $[CuO_2]^+$ and $[CuO]^+$ intermediates have low-lying singlet states, which are best calculated as an open-shell singlet with the broken-symmetry approach.[S22] All species with an even number of electrons have been calculated both as triplet and singlet species. The most stable state was employed, but small singlet–triplet splittings are commented. All reported energies were obtained from TPSS-D3 single-point calculations (with the full protein represented by

point-charges) with the def2-TZVPP basis set[S13] on structures obtained with TPSS-D3/def2-SV(P). In addition, we also report energies for similar calculations with TPSS-D3 replaced with B3LYP-D3.[S23–S25]

For selected states (**1**–**3**), we have probed the quality of the structures obtained with the TPSS-D3 functional and def2-SV(P) basis set by increasing the basis set to def2-TZVPD. In addition, test calculations were also performed with system 2 optimised. System 2 was defined as all atoms within 6 Å of any atom in system 1. We use the labels "free" and "fixed" for calculations in which the coordinates of atoms in system 2 were or were not optimized, respectively.

Overlay of active sites in 5ACF and 4EIS crystal structures

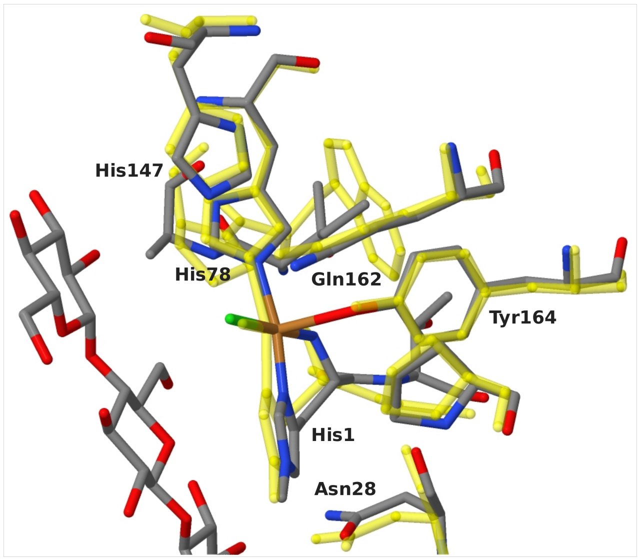

Figure S2: Overlay of active site structures from 5CAF and 4EIS (yellow). Hydrogen atoms are not shown. Residues numbering is accoriding to 5CAF.

Optimisations with system 2 free

Table S1: Cu–ligand bond lengths (Å) in the present and in previous studies for **3** with substrate (entries 1–3 and 9–11) and without substate (entries 4–8 and 12,13). All QM/MM results were obtained with the TPSS-D3 functional and def2-SV(P) basis set (unless otherwise noted).

Entry	Spin	Source	System 2	$Cu-N^{\epsilon}_{His78}$	$Cu-N_{His1}$	$Cu-N^{\epsilon}_{His1}$	$Cu-O_{Tyr}$	$Cu-O_2$	$O-H_1/O-H_4$
1	1	QM/MM	fixed	1.98	2.11	1.95	2.28	2.09	2.27/2.26
2	1	QM/MM	free	2.01	2.12	1.98	2.31	2.09	2.29/2.28
3	1[a]	QM/MM	free	2.00	2.12	1.97	2.39	2.07	2.35/2.41
4	1	QM/MM[S8]	fixed	2.06	2.15	2.01	2.89	2.04	-
5	1	QM/MM[S8]	free	2.06	2.13	2.00	2.84	2.04	-
6	1[a]	QM/MM[S8]	free	2.06	2.12	2.00	2.94	2.01	-
7	1[b]	QM/MM[S8]	free	2.08	2.11	2.01	2.84	1.99	-
8	1	QM-custer[S19]	-	1.98	2.09	1.97	3.35	1.98	-
9	1	QM-custer[S26]	-	1.97	2.16	1.95	4.14	2.11	3.02/2.38
10	0	QM/MM	fixed	1.99	2.11	1.96	2.28	2.06	2.24/2.25
11	0	QM/MM	free	2.01	2.11	1.99	2.31	2.06	2.26/2.27
12	0	QM/MM[S8]	fixed	2.06	2.15	2.01	2.87	2.02	-
13	0	QM/MM[S8]	free	2.06	2.12	2.00	2.82	2.03	-

[a] Optimised with TPSS-D3/def2-TZVPD [b] Optimised with B3LYP-D3/def2-TZVPD.

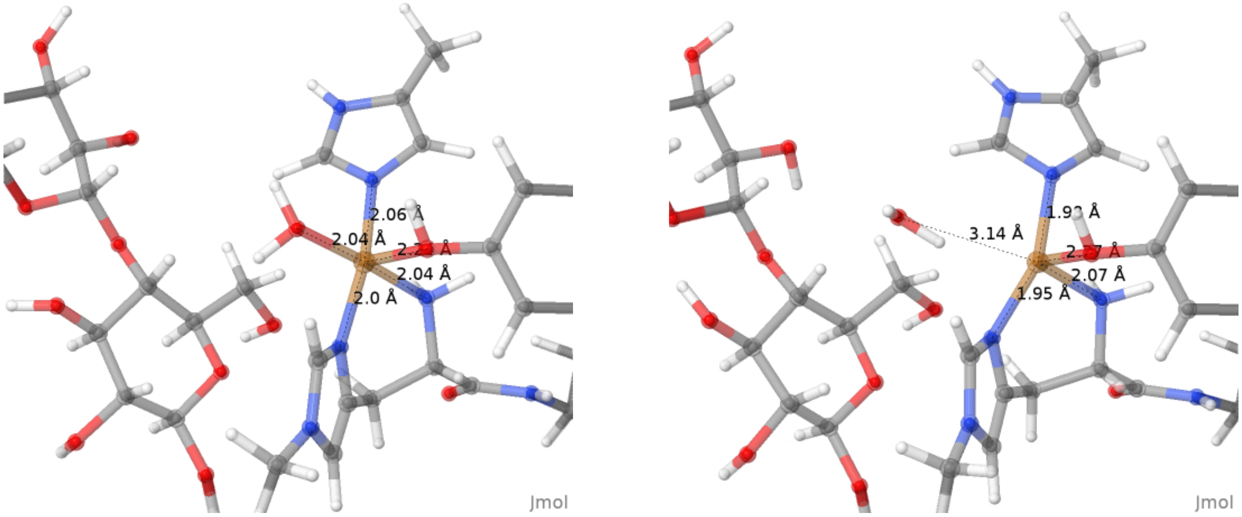

Figure S3: Optimised structures of $[Cu(H_2O)]^{2+}$ (**1**, *left*) and $[Cu(H_2O)]^{+}$ (**2**, *right*). The optimisation was carried out with TPSS-D3/def2-SV(P) with system 2 free.

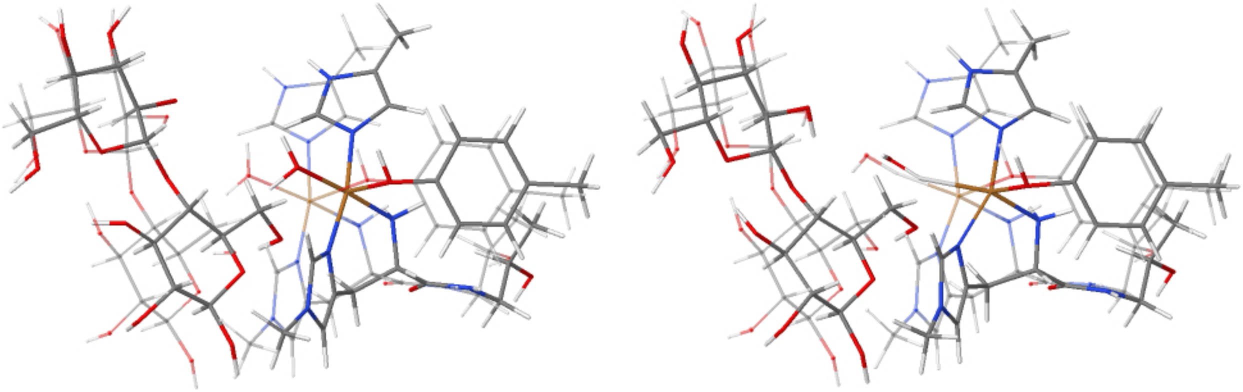

Figure S4: Comparison of optimised structures of $[Cu(H_2O)]^{2+}$ (**1**, *left*) and $[Cu(H_2O)]^{+}$ (**2**, *right*) with system 2 fixed (thin lines) or free (thick lines). The optimisations were carried out with TPSS-D3/def2-SV(P).

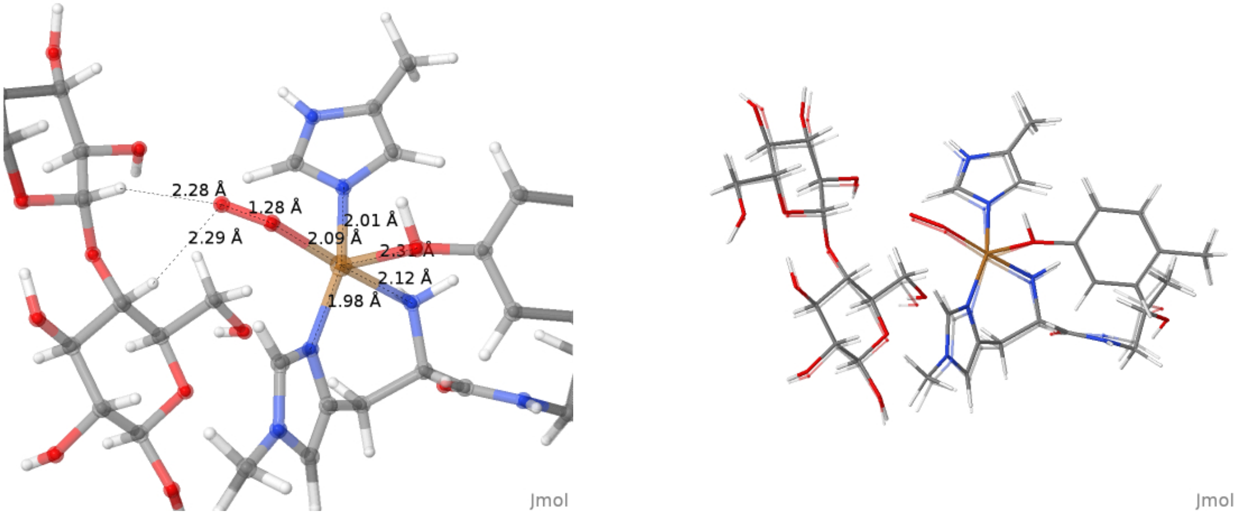

Figure S5: Optimised structure of **3**, obtained with TPSS-D3/def2-SV(P) and system 2 fixed (*left*). The figure to the *right* shows an overlay of **3** optimised with system 2 fixed (thick lines) and free (thin lines).

Singlet–triplet splittings of intermediate 3

Table S2: Singlet–triplet ($\Delta E = E_T - E_S$) splitting for **3** in kJ/mol in the present and in a previous study (obtained with TPSS-D3/def2-SV(P) without His147 in the QM region). In addition, the QM/MM energy components from Eqn. 1 are also included (with $\Delta E_{\mathrm{MM}} = \Delta E_{\mathrm{MM123}} - \Delta E_{\mathrm{MM1}}$), as well as the QM+ptch energy calculated with the larger def2-TZVPP basis set (single-point energy calculation; ΔE_{QM}).

Source	System 2	Func	$\Delta E_{\mathrm{QM/MM}}$	$\Delta E_{\mathrm{QM+ptch}}$	ΔE_{QM}	ΔE_{MM}
QM/MM[S8]	free	TPSS-D3	12.5	10.6	14.0	2.0
QM/MM[S8]	free	B3LYP-D3	13.2	11.2	16.5	2.0
QM/MM	free	TPSS-D3	11.9	10.3	14.2	1.5
QM/MM	free	B3LYP-D3	14.0	12.4	17.0	1.5

Reaction profiles and energetics for intermediates 3–7

Table S3: Reaction energies and barriers (kJ/mol) obtained from single-point calculations with the def2-TZVPP basis set (for the meaning of the different energies, see Models and Methods section of the main paper).

Reaction	**3→4a**			
ΔE	TPSS-D3		B3LYP-D3	
Struct	TS	Prod	TS	Prod
$\Delta E_{\mathrm{QM/MM}}$	14.9	-5.6	17.0	0.1
$\Delta E_{\mathrm{QM+ptch}}$	17.8	-3.6	19.9	2.0
ΔE_{MM}	-2.9	-1.9	-2.9	-1.9
ΔE_{QM}	24.5	52.1	27.2	63.9
ΔE_{ptch}	-6.7	-55.7	-7.3	-61.8

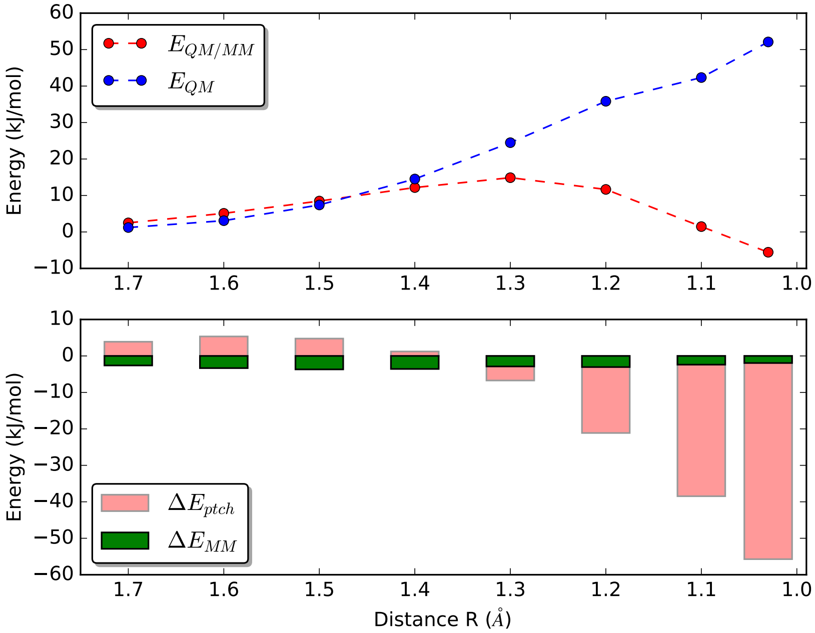

Figure S6: $\Delta E_{\mathrm{QM/MM}}$ and ΔE_{QM} for a range of O−H distances (reaction **3**→**4a**). In the lower figure, the electrostatic and MM contributions to $\Delta E_{\mathrm{QM/MM}}$ are shown for the various distances.

Table S4: Reaction energies and barriers (kJ/mol) obtained from single-point calculations with the def2-TZVPP basis set (for the meaning of the different energies, see Models and Methods section of the main paper).

Reaction	**4b**→**6a**			
ΔE	TPSS-D3		B3LYP-D3	
Energy	TS	Prod	TS	Prod
$\Delta E_{\mathrm{QM/MM}}$	47.8	-0.5	66.2	54.1
$\Delta E_{\mathrm{QM+ptch}}$	54.7	1.7	68.5	56.3
ΔE_{MM}	-6.9	-2.2	-6.9	-2.2
ΔE_{QM}	110.5	98.2	165.6	157.8
ΔE_{ptch}	-55.8	-96.4	-97.1	-101.5

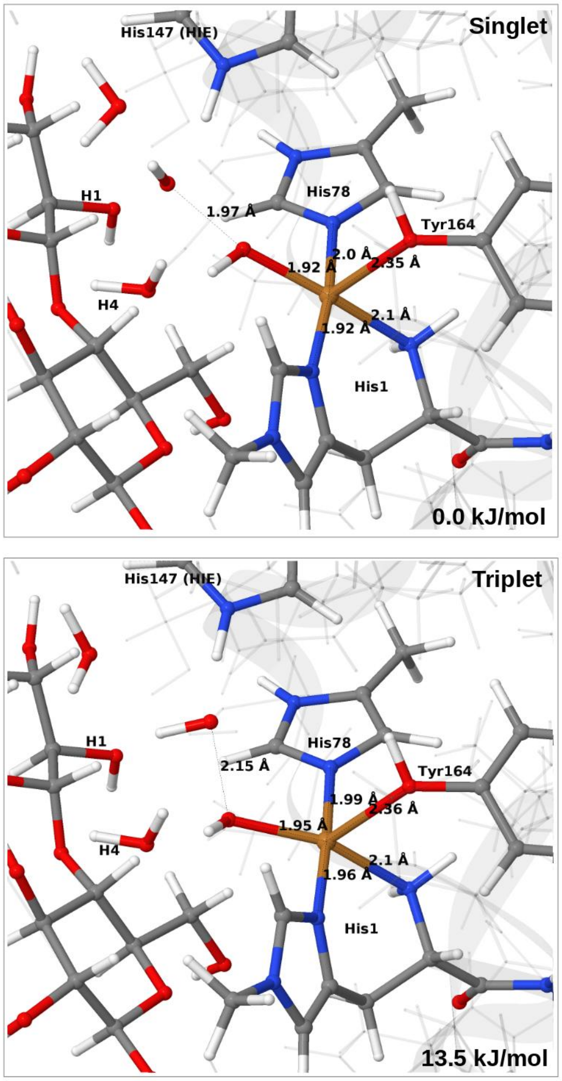

Figure S7: Optimized structures of state **5** with HIE147 in the singlet (*top*) and triplet (*bottom*) spin-states.

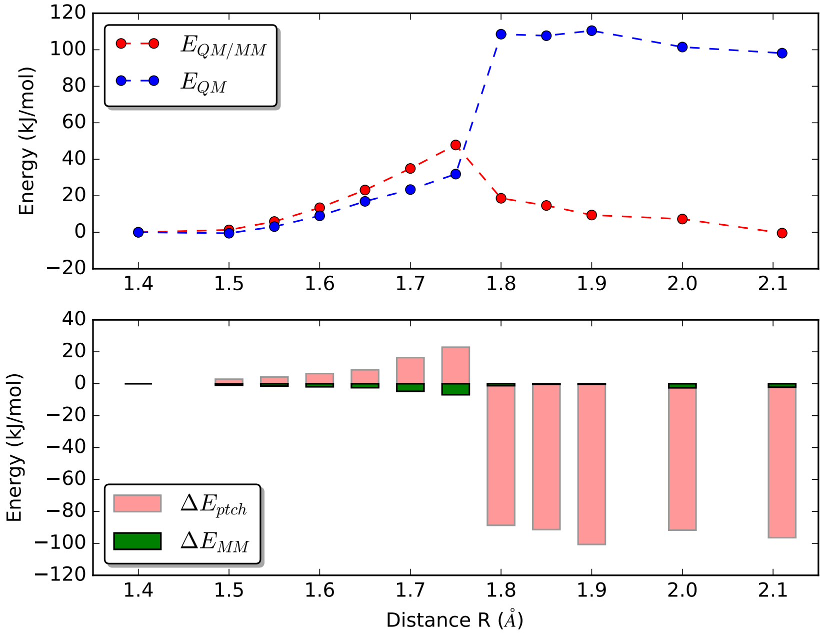

Figure S8: $\Delta E_{\mathrm{QM/MM}}$ and ΔE_{QM} for a range of O−O distances in the water dissociation reaction **4b**→**6a**. In the lower figure, the electrostatic and MM contributions to $\Delta E_{\mathrm{QM/MM}}$ are shown for the various distances.

Table S5: Reaction energies and barriers obtained with def2-TZVPP singlet-point calculations. His147 was modelled as HIE.

Reaction	C_4−H abstraction by **3**			
ΔE	TPSS-D3		B3LYP-D3	
Method	TPSS-D3		B3LYP-D3	
$\Delta E_{\mathrm{QM/MM}}$	155.7	140.3	168.3	128.0
$\Delta E_{\mathrm{QM+ptch}}$	154.5	135.6	167.1	123.2
ΔE_{MM}	1.1	4.7	1.1	4.7
ΔE_{QM}	151.5	133.5	162.2	123.6
ΔE_{ptch}	3.0	2.1	4.9	-0.4

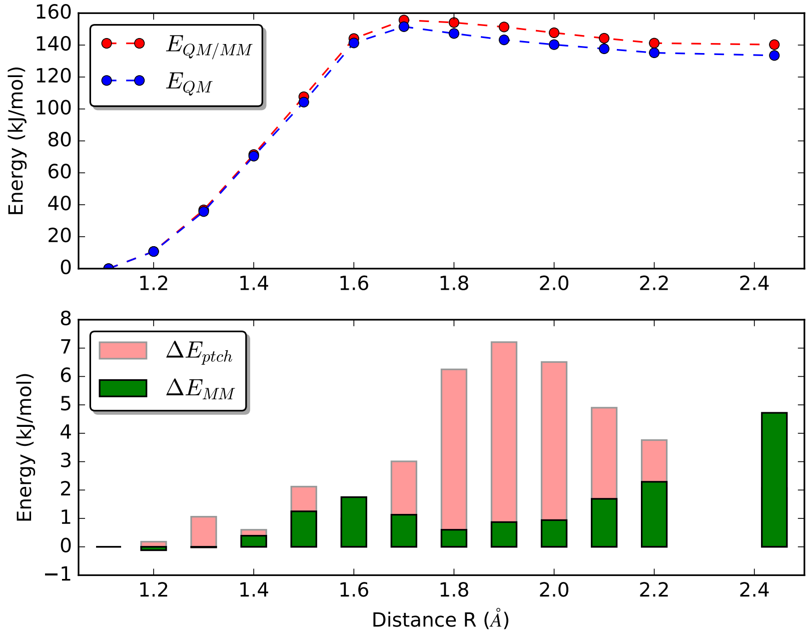

Figure S9: Reaction energies and barrier for C−H hydrogen abstraction by $[CuO_2]^+$. The figure shows $\Delta E_{\mathrm{QM/MM}}$ and ΔE_{QM} for a range of O−H distances. In the lower figure, the electrostatic and MM contributions to $\Delta E_{\mathrm{QM/MM}}$ are shown for the various distances.

Table S6: Reaction energies and barriers for the hydrogen abstraction from RH by the $[CuO]^+$ (**6b**) and $[CuOH]^{2+}$ (**6c**) states. The energies are obtained with def2-TZVPP singlet-point calculations.

Reaction	**6c**→**7b** (HID147)				**6b**→**7a** (HID147)			
ΔE	TPSS-D3		B3LYP-D3		TPSS-D3		B3LYP-D3	
	TS	Prod	TS	Prod	TS	Prod	TS	Prod
$\Delta E_{\mathrm{QM/MM}}$	93.8	48.5	97.0	-22.2	68.5	-17.2	72.9	-21.8
$\Delta E_{\mathrm{QM+ptch}}$	75.8	31.4	84.2	-39.4	67.0	-23.7	71.3	-28.4
ΔE_{MM}	12.8	17.2	12.8	17.2	1.6	6.6	1.6	6.6
ΔE_{QM}	76.7	44.8	82.8	-3.9	68.2	-12.3	73.9	-16.8
ΔE_{ptch}	-2.8	-13.5	1.4	-35.5	-1.2	-11.4	-2.6	-11.6

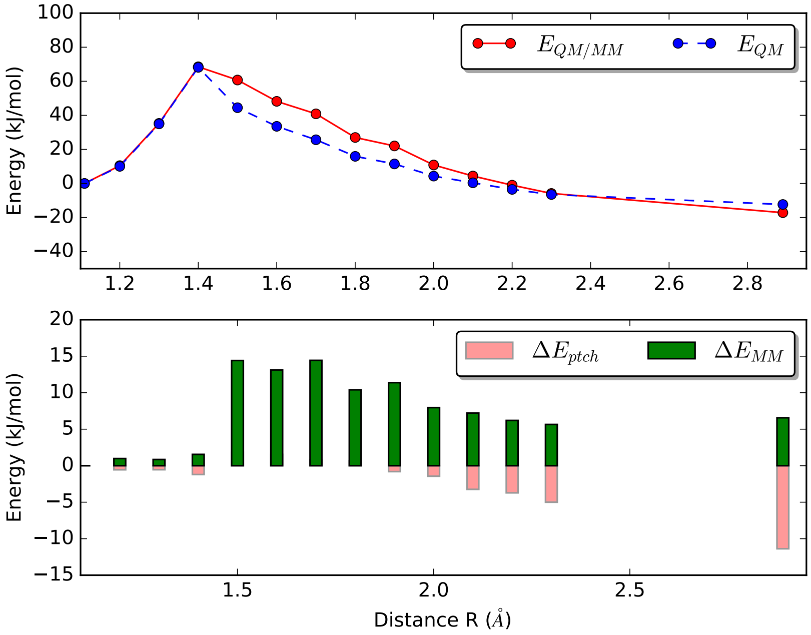

Figure S10: Reaction energies and barriers for C−H hydrogen abstraction by $[CuO]^+$ (**6b**). The figure shows $\Delta E_{\mathrm{QM/MM}}$ and ΔE_{QM} for a range of C_4−H distances. In the lower figure, the electrostatic and MM contributions to $\Delta E_{\mathrm{QM/MM}}$ are shown for the various distances.

Table S7: Reaction energies and barriers for hydrogen abstraction from RH by the $[CuO]^+$ (**6b**) and $[CuOH]^{2+}$ (**6c**) moieties. The energies are obtained with def2-TZVPP singlet-point calculations.

Reaction	**6c**→**7b** (HIE147)				**6b**→**7a** (HIE147)			
ΔE	TPSS-D3		B3LYP-D3		TPSS-D3		B3LYP-D3	
	TS	Prod	TS	Prod	TS	Prod	TS	Prod
$\Delta E_{\mathrm{QM/MM}}$	103.4	56.7	92.9	-13.5	104.5	-22.4	111.1	-27.1
$\Delta E_{\mathrm{QM+ptch}}$	85.5	39.9	92.9	-30.3	102.4	-25.4	109.0	-30.1
ΔE_{MM}	17.9	16.8	0.0	16.8	2.2	3.0	2.2	3.0
ΔE_{QM}	94.2	59.4	-	-14.8	100.9	-20.1	112.3	-24.8
ΔE_{ptch}	-8.7	-19.5	-	-15.6	1.5	-5.4	-3.3	-5.3

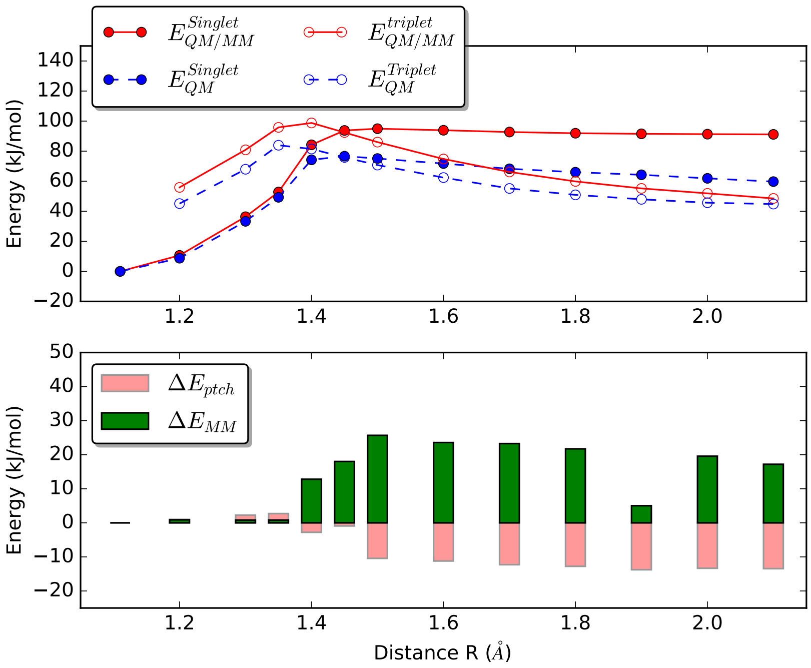

Figure S11: Reaction energies and barriers for C−H hydrogen abstraction by $[CuOH]^{2+}$ (**6c**). The figure shows $\Delta E_{\mathrm{QM/MM}}$ and ΔE_{QM} for a range of O−H distances. In the lower figure, the electrostatic and MM contributions to $\Delta E_{\mathrm{QM/MM}}$ are shown for the various distances (always for the most stable state, singlet or triplet).

Table S8: Reaction energies and barriers (kJ/mol) for the recombination step (**7a**→**8a**) with His147 in either the HIE or the HID state. The energies are obtained from single-point calculations with the def2-TZVPP basis set.

Reaction	**7a**→**8a** (HIE)				**7a**→**8a** (HID)			
ΔE	TPSS-D3		B3LYP-D3		TPSS-D3		B3LYP-D3	
Energy	TS	Prod	TS	Prod	TS	Prod	TS	Prod
$\Delta E_{\mathrm{QM/MM}}$	40.3	-182.6	53.4	-193.1	35.1	-194.0	44.3	-203.8
$\Delta E_{\mathrm{QM+ptch}}$	30.0	-168.6	31.5	-205.1	23.2	-218.5	22.9	-228.3
ΔE_{MM}	10.3	12.0	21.9	12.0	11.9	24.5	21.4	24.5
ΔE_{QM}	38.8	-154.3	36.6	-190.2	30.1	-203.5	21.2	-213.0
ΔE_{ptch}	-8.8	-14.4	-5.2	-15.0	-6.9	-14.4	1.8	-15.3

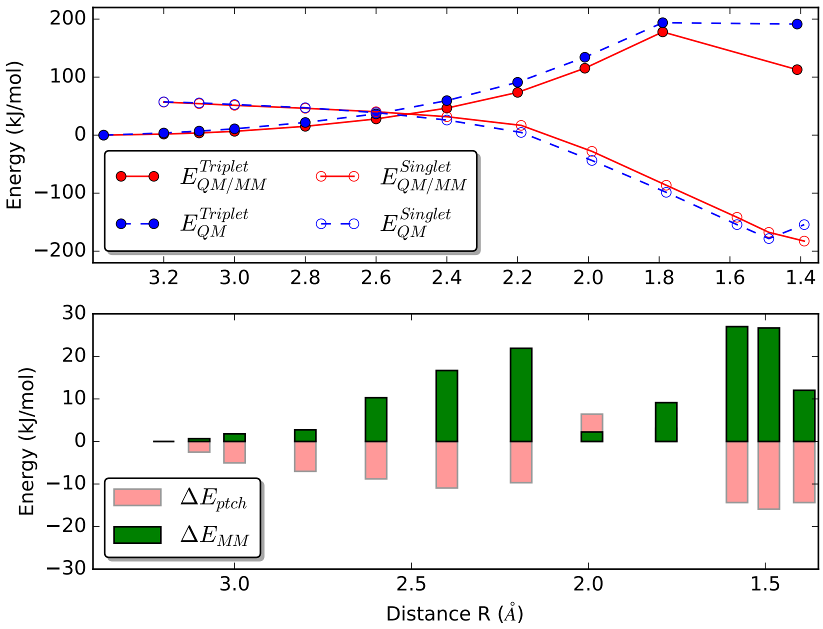

Figure S12: Reaction energies and barrier for the recombination step (**7a**→**8a**) with His147 in the HIE state. The figure shows $\Delta E_{\mathrm{QM/MM}}$ and ΔE_{QM} for a range of C−O distances. In the lower figure, the electrostatic and MM contributions to $\Delta E_{\mathrm{QM/MM}}$ are shown for the various distances.

Table S9: Reaction energies and barriers (kJ/mol) obtained for the recombination step (**7a**→**8a**) with His147 in the HIP state. The energies are obtained from single-point calculations with the def2-TZVPP basis set .

Reaction	**7a**→**8a** (HIP)			
ΔE	TPSS-D3		B3LYP-D3	
Energy	TS	Prod	TS	Prod
$\Delta E_{\mathrm{QM/MM}}$	61.9	-158.1	53.1	-165.7
$\Delta E_{\mathrm{QM+ptch}}$	51.5	-165.7	48.7	-156.8
ΔE_{MM}	47.5	-156.8	4.4	-163.1
ΔE_{QM}	10.5	7.6	28.1	7.6
ΔE_{ptch}	3.9	-8.8	20.5	-7.0

References

(S1) Frandsen, K. E. H.; Simmons, T. J.; Dupree, P.; Poulsen, J.-C. N.; Hemsworth, G. R.; Ciano, L.; Johnston, E. M.; Tovborg, M.; Johansen, K. S.; von Freiesleben, P.; Marmuse, L.; Fort, S.; Cottaz, S.; Driguez, H.; Henrissat, B.; Lenfant, N.; Tuna, F.; Baldansuren, A.; Davies, G. J.; Leggio, L. L.; Walton, P. H. *Nat. Chem. Biol.* **2016**, *12*, 298–305.

(S2) Sommerhalter, M.; Lieberman, R. L.; Rosenzweig, A. C. *Inorg. Chem.* **2005**, *44*, 770–778.

(S3) Hemsworth, G. R.; Taylor, E. J.; Kim, R. Q.; Gregory, R. C.; Lewis, J. P., S. J. Turkenburg; Parkin, A.; Davies, G. J.; Walton, P. H. *J. Am. Chem. Soc.* **2013**, *135*, 6069–6077.

(S4) Gudmundsson, M.; Kim, S.; Wu, M.; Ishida, T.; Momeni, M. H.; Vaaje-kolstad, G.; Lundberg, D.; Royant, A.; Ståhlberg, J.; Eijsink, V. G. H.; Beckham, G. T.; Sandgren, M. *J. Biol. Chem.* **2014**, *289*, 18782–18792.

(S5) Maestro version 10.2 (2015). Schrödinger, LLC, New York, NY, 2015.

(S6) Olsson, M. H. M.; Søndergard, C. R.; Rostkowski, M.; Jensen, J. H. *J. Chem. Theory Comput.* **2011**, *7*, 525–537.

(S7) Dell, W. B. O.; Agarwal, P. K.; Meilleur, F. *Angew. Chem. Int. Ed.* **2017**, *56*, 767–770.

(S8) Hedegård, E. D.; Ryde, U. *ACS Omega* **2017**, *2*, 536–545 (arXiv:1612.05513).

(S9) Kirschner, K. N.; Yongye, A. B.; Tschampel, S. M.; González-Outeiriño, J.; Daniels, C. R.; Foley, B. L.; Woods, R. J. *J. Comput. Chem.* **2008**, *29*, 622–655.

(S10) Maier, J. A.; Martinez, C.; Kasavajhala, K.; Wickstrom, L.; Hauser, K. E.; Simmerling, C. *Journal of Chemical Theory and Computation* **2015**, *11*, 3696–3713.

(S11) Jørgensen, P., P. Swanstrøm; Yeager, D. L. *J. Chem. Phys.* **1983**, *78*, 347–356.

(S12) Tao, J.; Perdew, J. P.; Staroverov, V. N.; Scuseria, G. E. *Phys. Rev. Lett.* **2003**, *91*, 146401.

(S13) Schäfer, A.; Horn, H.; Ahlrichs, R. *J. Chem. Phys.* **1992**, *97*, 2571–2577.

(S14) Eichkorn, K.; Weigend, F.; Treutler, O.; Ahlrichs, R. *Theor. Chem. Acc.* **1997**, *97*, 119–124.

(S15) Singh, C. A.; Kollman, P. A. *J. Comp. Chem.* **1984**, *5*, 129–45.

(S16) Besler, B. H.; Merz Jr., K. M.; Kollman, P. A. *J. Comp. Chem.* **1990**, *11*, 431–439.

(S17) Sigfridsson, E.; Ryde, U. *J. Comp. Chem.* **1998**, *19*, 377–395.

(S18) Kim, S.; Ståhlberg, J.; Sandgren, M.; Patond, R. S.; Beckham, G. T. *Proc. Natl. Sci. USA* **2014**, *111*, 149–154.

(S19) Kjaergaard, C. H.; Qayyum, M. F.; Wong, S. D.; Xu, F.; Hemsworth, G. R.; Walton, D. J.; Solomon, E. I. *Proc. Natl. Acad. Sci. USA* **2014**, *111*, 8797–8802.

(S20) Grimme, S.; Antony, J.; Ehrlich, S.; Krieg, H. *J Chem. Phys.* **2010**, *132*, 154104.

(S21) Grimme, S.; Ehrlich, S.; Goerigk, L. *J. Comput. Chem.* **2011**, 1456–1465.

(S22) Noodleman, L.; Davidson, E. R. *Chem. Phys.* **1986**, *109*, 131–143.

(S23) Becke, A. D. *Phys. Rev. A* **1988**, *38*, 3098–3100.

(S24) Becke, A. D. *J. Chem. Phys.* **1993**, *98*, 5648–5652.

(S25) Lee, C.; Yang, W.; Parr, R. G. *Phys. Rev. B* **1988**, *37*, 785–789.

(S26) Bertini, L.; Lambrughi, M.; Fantucci, P.; De Gioia, L.; Borsari, M.; Sola, M.; Bortolotti, C. A.; Bruschi, M. *Inorg. Chem.* **2017**, in press.
